# Oral administration of kratom leaf extract alleviates anxiety-like behavior, urinary bladder pain, voiding dysfunction, and bladder hypercontractility via attenuating muscarinic receptor response in male mice exposed to chronic water avoidance stress

**DOI:** 10.3389/fnins.2026.1810337

**Published:** 2026-04-22

**Authors:** Sarunnuch Sattayachiti, Panida Chumpong, Dania Cheaha, Nattapon Rotpenpian, Ekkasit Kumarnsit, Nipaporn Konthapakdee

**Affiliations:** 1 Division of Health and Applied Sciences, Faculty of Science, Prince of Songkla University, Songkhla, Thailand; 2 Division of Biological Science, Faculty of Science, Prince of Songkla University, Songkhla, Thailand; 3 Biosignal Research Center for Health, Faculty of Science, Prince of Songkla University, Songkhla, Thailand; 4 Department of Oral Biology and Occlusion, Faculty of Dentistry, Prince of Songkla University, Songkhla, Thailand

**Keywords:** anxiety-like behaviors, bladder hyperactivity, bladder pain, kratom leaf extract, mitragynine, psychological stress, voiding pattern analysis, water avoidance stress

## Abstract

Psychological stress causes and deteriorates interstitial cystitis/painful bladder syndrome with urinary frequency, incontinence, bladder pain and urgency. The major alkaloid of kratom (*Mitragyna speciosa*), mitragynine, shows analgesic, anxiolytic, and smooth muscle relaxant effects. However, the effects of kratom leaf extract on stress-induced anxiety-like behavior, urinary bladder pain and urinary bladder dysfunction remain unknown. Therefore, this study aims to examine the effect of kratom leaf extract administration on anxiety-like behaviors, bladder pain, bladder contractile properties, and mast cell number in mice exposed to water avoidance stress. Male C57BL/6 mice were exposed to water avoidance stress (WAS) protocol for 10 consecutive days and compared with the stress-exposed mice receiving oral administration of kratom leaf extract (2.5 and 5 mg/kg of mitragynine) or solifenacin (10 mg/kg). Anxiety-like behaviors were assessed using open field test. Bladder pain sensitivity was evaluated with von Frey test, while voiding behavior was analyzed using voiding pattern analysis. Bladder contractility was examined using an *in vitro* organ bath technique, and urinary bladder mast cell infiltration was assessed by toluidine blue staining. Results show that mice receiving WAS had a reduction in the total duration and number of unsupported rearing behaviors, reduced voiding area, and increased bladder pain responses; however, these effects were reversed by treatment with kratom leaf extract (2.5 and 5 mg/kg of mitragynine). Interestingly, the WAS group also exhibited markedly increased tonic contractions in response to carbachol, a muscarinic agonist; these responses were attenuated in mice treated with kratom leaf extract (2.5 and 5 mg/kg) The enhanced tonic contractile response to carbachol was abolished by pre-incubation with ondansetron (a 5-HT₃ antagonist). The WAS group showed an increased total number of mast cells in the urinary bladder, which was reduced by treatment with kratom leaf extract at both 2.5 and 5 mg/kg. Our results indicate that treatment with kratom leaf extract attenuated chronic stress–induced bladder pain responses, voiding abnormalities, and mast cell numbers, and was associated with reduced contractile response to muscarinic stimulation, suggesting a potential modulatory effect on stress-induced bladder dysfunction.

## 1 Introduction

Urinary bladder dysfunctions, including overactive bladder (OAB) and interstitial cystitis/painful bladder syndrome (IC/PBS), are prevalent and debilitating disorders of the lower urinary tract (Rothrock et al., 2001; Leron et al., 2018; Yu et al., 2026). IC/PBS patients commonly experience urinary frequency, urgency, incontinence, nocturia, and pelvic pain, thereby impairing daily functioning and social performance, including work, sleep, physical activity, and sexual function (Rothrock et al., 2001; Miwa et al., 2016; Leron et al., 2018). Increasing clinical evidences indicates that psychological stress triggers and exacerbates lower urinary tract symptoms, including OAB and IC/PBS (Rothrock et al., 2001). Mechanisms of stress induced urinary bladder dysregulation involve activation of the hypothalamic–pituitary–adrenal (HPA) axis, autonomic imbalance, and increased sympathetic outflow, which contribute to altered bladder afferent signaling, detrusor overactivity, and urothelial dysfunction (Nargund, 2015; Matos et al., 2017; Gao and Rodríguez, 2022).

Water avoidance stress (WAS) is a widely used rodent paradigm that has been shown to induce anxiety-like behaviors and bladder dysfunction, and is therefore commonly employed to study stress-related bladder dysfunction (Smith et al., 2011; Mills et al., 2021; West et al., 2021a; Sattayachiti et al., 2024; Waemong et al., 2024). Previous studies have commonly implemented a 10-day WAS protocol to model chronic psychological stress in rodents, demonstrating that this duration is sufficient to mimic stress-associated urinary bladder dysfunction (Smith et al., 2011; West et al., 2021a; Sattayachiti et al., 2022). Chronic WAS exposure for 10 days has been shown to induce bladder dysfunction, characterized by marked alterations in voiding patterns, including increased urinary frequency, a higher number of small voids, shortened inter-micturition intervals, and reduced average void size (West et al., 2021b; Sattayachiti et al., 2022; West et al., 2022a). In addition to voiding abnormalities, prolonged WAS exposure induces persistent bladder hyperalgesia, elevates urinary noradrenaline levels, enhances suprapubic pain sensitivity, and causes histopathological alterations in the bladder, including urothelial disruption and mast cell infiltration (Lee et al., 2015; Matos et al., 2017). Furthermore, our recent findings indicate that serotonergic signaling mediated through 5-HT_3_ receptors contributes to stress-induced bladder dysfunction, as pharmacological blockade with the selective 5-HT_3_ receptor antagonist, ondansetron, improved voiding patterns and attenuated the enhanced muscarinic responsiveness observed in mice subjected to a 10-day WAS protocol (Sattayachiti et al., 2022).

Despite advances in understanding stress-related bladder pathophysiology, current treatment options remain limited. Standard therapies mainly involve antimuscarinic agents (e.g., oxybutynin, tolterodine, solifenacin); however, their clinical use is constrained by side effects such as dry mouth, constipation, blurred vision, and drowsiness (Davila et al., 2001; Abraham and Goldman, 2015). Furthermore, these drugs primarily suppress detrusor muscle contractions without addressing the central or systemic mechanisms underlying stress-related bladder symptoms. These limitations highlight the need for alternative therapies with neuromodulatory, anxiolytic, and smooth muscle-modulating properties that can provide effective symptom relief.

Kratom, *Mitragyna speciosa*, is an indigenous perennial plant of Southeast Asia. In Thailand, kratom was officially removed from the list of narcotics in 2021 and is currently regulated under the Kratom Plant Act, 2565 B.E. (2022 CE) (2022), which allows its use for medicinal research and as a dietary supplement (Kratom Plant Act, 2565 B.E. (2022 CE), 2022). Kratom has attracted growing scientific interest due to its diverse pharmacological effects, including pain relief, antidiarrheal activity, treatment of gastrointestinal disorders, mood enhancement, and increased stamina (Adkins et al., 2011; Brown et al., 2017; Annuar et al., 2024). Notably, recent *in vivo* studies have demonstrated that kratom extract improves colitis symptoms, gastrointestinal transit, and intestinal barrier integrity in colitis-induced mice (Sengnon et al., 2026). Kratom leaf extract contains several bioactive alkaloids, including mitragynine, 7-hydroxymitragynine, and paynantheine, with mitragynine being the predominant constituent, accounting for up to approximately 66% of the total alkaloid content (Takayama, 2004; Swogger et al., 2022). Interestingly, previous studies have reported that mitragynine, a major substance in kratom leaf, produces anxiolytic and antidepressant-like effects by modulating neurotransmitter levels, including serotonin, noradrenaline, and dopamine, in brain regions involved in the regulation of mood and anxiety (Johnson et al., 2020; Manus et al., 2025). Administration of kratom alkaloid extract reduces immobility in the forced swim test (Kumarnsit et al., 2007), and mitragynine has also been shown to lower corticosterone levels in mice subjected to forced swim and tail suspension tests (Idayu et al., 2011). In addition, mitragynine exerts antinociceptive effects by binding to and activating supraspinal *μ*- and *δ*-opioid receptors, thereby inhibiting the transmission of pain signals in sensory neurons (Thongpradichote et al., 1998; Ramachandram et al., 2020). Unlike traditional opioids such as morphine, mitragynine preferentially stimulates the G-protein signaling with minimal recruitment of *β*-arrestin 2, which may reduce opioid-related adverse effects, including respiratory depression, constipation, and addiction (Boyer et al., 2008; Prozialeck et al., 2012; Váradi et al., 2016; Alford et al., 2025).

Furthermore, experimental evidence demonstrates that mitragynine inhibits electrically induced contractions of the guinea pig ileum in a concentration-dependent manner, suggesting reduced smooth muscle contraction via suppression of neurotransmitter release at nerve terminals (Watanabe et al., 1997). Crude methanolic extracts of kratom decrease intestinal motility and alleviate castor oil-induced diarrhea in rats; this effect is not reversed by naloxone, indicating mechanisms beyond *μ*-opioid receptors or combined actions of multiple alkaloids (Chittrakarn et al., 2008). A recent finding also suggests that kratom extract modulates gut microbiota composition, increasing beneficial bacterial populations (Thongsepee et al., 2025). Collectively, this evidence highlight that kratom exerts multifaceted central and peripheral effects, including analgesic, anxiolytic, and smooth muscle-relaxant effects on the gastrointestinal tract, an organ closely associated with the urinary bladder. However, the effects of kratom leaf extract on urinary bladder function, particularly in the context of stress-related bladder dysfunction and bladder pain, remain unexplored. Therefore, the present study aimed to investigate the effects of kratom leaf extract on anxiety-related behaviors, voiding patterns, urinary bladder pain, bladder contractility, and mast cell infiltration in the bladders of mice subjected to chronic water avoidance stress. Findings from this study will provide novel insights into the therapeutic potential of kratom in stress-related bladder symptoms, e.g., OAB and IC/PBS.

## 2 Materials and methods

### 2.1 Preparation of kratom leaf extract and phytochemical characterization

Fresh kratom leaves (*Mitragyna speciosa*) were collected from Na San District, Surat Thani Province, Thailand. The leaves were washed, air-dried under sunlight until completely dry, and ground into a fine powder. Approximately 250–300 grams of the powdered leaves were extracted with 2 liters of Milli-Q water by boiling in a high-pressure cooker (800 W) for 3 min. The mixture was filtered to remove plant residues. The remaining solid residue was re-extracted with an additional 2 liters of Milli-Q water using the same boiling conditions. The resulting liquid extracts were combined and stored at 4 °C. The extract was subsequently centrifuged at 10,000 rpm for 3 min to eliminate residual particulates. The supernatant was filtered again and subsequently subjected to freeze-drying to yield the extract in powdered form.

**Figure 1:**
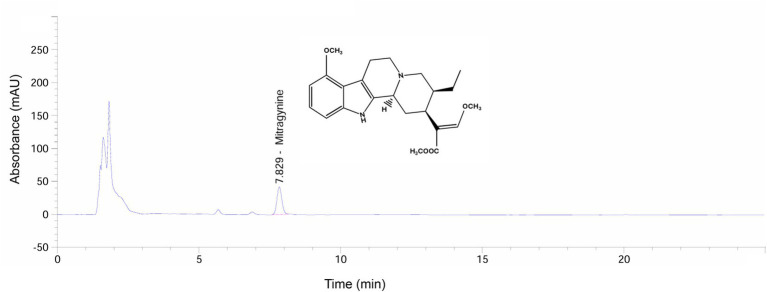
High-performance liquid chromatography (HPLC) chromatogram of mitragynine in the kratom leaf extract sample.

The powdered kratom leaf extract was analyzed for mitragynine content using high-performance liquid chromatography (HPLC), conducted by the Office of Scientific Instrument and Testing (OSIT), Prince of Songkla University, Songkhla, Thailand. The peak in the chromatogram exhibited an identical retention time of 7.829 min (Figure 1), corresponding to that of the mitragynine standard, confirming its identity. The area under the peak was used to calculate the concentration of mitragynine in the sample based on the standard calibration curve. As a result, the kratom extract used in this study was found to contain approximately 24.46 ± 2.37 mg of mitragynine per gram of extract.

### 2.2 Experimental animals and treatments

Adult male C57BL/6NJcl mice (8–10 weeks old) were purchased from Nomura Siam Co., Ltd. and housed individually in a temperature-controlled room (25 °C ± 2 °C) under a 12:12-h light–dark cycle. All mice were acclimatized for at least 1 week prior to the experiments and were provided with free access to drinking water and a standard diet (S.W.T., Thailand). All experiments were conducted with the approval of the Animal Ethics Committee at Prince of Songkla University, Thailand (protocol code 2023-SCI07-012).

Mice were randomly divided into five groups: control (no stress protocol), water avoidance stress (WAS), water avoidance stress treated with kratom leaf extract containing 2.5 mg/kg of mitragynine (WAS+2.5KT), water avoidance stress treated with kratom leaf extract containing 5 mg/kg of mitragynine (WAS+5KT), and water avoidance stress treated with 10 mg/kg solifenacin (WAS+Soli) groups. Body weight of each mouse was measured daily throughout the experimental period.

**Figure 2:**
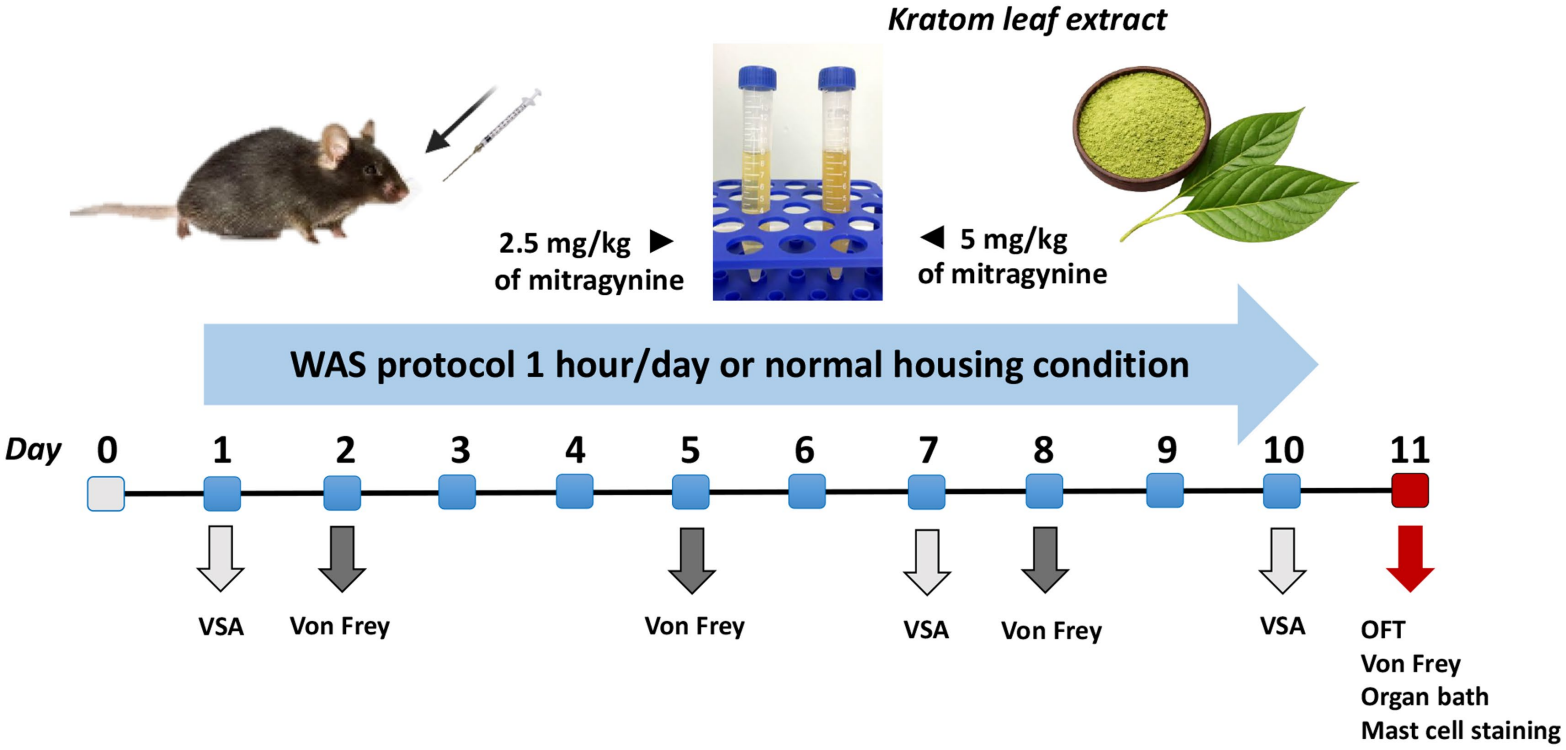
Overview schematic of the experimental design of this study.

The treatments were administered once daily, 1 h before exposure to WAS for 10 consecutive days at doses of of 2.5 mg/kg BW and 5 mg/kg BW before the water avoidance stress protocol. The doses of kratom leaf extract for the 2.5KT and 5KT groups were based on the amount of mitragynine in the extract at 2.5 mg/kg body weight (BW)/day and 5 mg/kg BW/day, respectively. Mice in WAS+Soli group were orally gavaged with 10 mg/kg BW of solifenacin [Astellas Pharma (Thailand) Co., Ltd., Bangkok, Thailand] as the positive control group in accordance with previously established protocols (Chittrakarn et al., 2008). Meanwhile, mice in the control and WAS groups received oral administration of distilled water as a vehicle for the kratom leaf extract and solifenacin. An overview schematic diagram of this study is shown in Figure 2.

### 2.3 Water avoidance stress (WAS)

Water avoidance stress was conducted in this study following previously described protocols (West et al., 2021a; Sattayachiti et al., 2024). Mice in all groups, except the control group, were exposed daily for 10 consecutive days. During each session, mice were individually placed on a cylindrical platform at the center of a polypropylene box, which was surrounded by room-temperature water, for 1 h per day (10.00–11.00 a.m.). After each WAS exposure, the animals were returned to their home cages. Animals in the control group remained in their standard housing conditions throughout the experiment.

### 2.4 Voiding spot analysis (VSA)

Urination patterns of each group were assessed using voiding spot analysis after WAS exposure on days 1, 7, and 10. Each mouse was placed on a wire mesh floor inside a standard cage, with filter paper (Whatman™ Grade 1, Cat. No. 17023133) positioned beneath to collect urine over a four-hour period from 12.00 a.m. to 4.00 p.m. Water was withheld during the testing period to minimize the risk of urine spot dilution or spreading caused by dripping water, but freely access to food. Following the collection period, the filter papers were air-dried and photographed under UV light to visualize the urine patterns. The number and area of urine spots on the filter paper were then photographed and quantified using ImageJ software. Only urine spots with an area ≥ 0.4 cm^2^ were considered eligible for inclusion in the analysis, while those with an area < 0.4 cm^2^ were excludsed. N number: Control = 8; WAS = 8; WAS+2.5KT = 8; WAS+5KT = 8; Soli = 6.

### 2.5 Open field test (OFT)

One day after the final WAS session, an open field test was conducted to assess locomotor activity and anxiety-like behaviors of mice from all experimental groups. The OFT experiment was conducted following a previously established protocol (Waemong et al., 2024). Each mouse was placed in a white plastic enclosure with dimensions of 15 × 12 × 12 inches (length × width × height) and allowed to explore freely for 15 min. A video camera was mounted above the enclosure to record movements and behaviors throughout the session. Locomotor activity was analyzed using OptiMouse MATLAB software (Ben-Shaul, 2017), which tracked the mouse’s movements by contrasting their dark bodies with the bright background. Anxiety-related behaviors were also evaluated by an observer based on three main criteria: (1) grooming, defined as self-cleaning actions (licking or rubbing of the paws, head, or body) lasting longer than 2 s; (2) unsupported rearing, defined as mice standing upright on their hind limbs without contacting the enclosure walls for more than 1 s; and (3) supported rearing, characterized by mice standing on their hind limbs while bracing against the enclosure walls for more than 1 s. N number: Control = 7; WAS = 6; WAS+2.5KT = 7; WAS+5KT = 6; Soli = 5.

### 2.6 Von Frey test

After completing the open field test, all mice underwent assessment of nociceptive-like behavior associated with bladder pain using von Frey filaments. Mice were placed individually in a small acrylic box with penetrable wire mesh floor and were allowed to acclimate to the experimental environment for at least 10 min. Mechanical allodynia in the lower abdominal area, corresponding to the urinary bladder area, was evaluated using von Frey filaments with forces of 0.04, 0.16, 0.4, 1.0, and 4.0 grams. Each filament was applied 10 times, with a 5-s interval between applications. Nociceptive responses were scored as follows: (0), no response; (1), sudden abdominal retraction; (2), immediate licking or scratching of the stimulated area; or (3), change in position or jumping (Rudick et al., 2008). N number: Control = 6; WAS = 6; WAS+2.5KT = 4; WAS+5KT = 4; Soli = 5.

### 2.7 Organ bath studies

On day 11, mice were anesthetized with thiopental sodium (70 mg/kg, intraperitoneally). Following confirmation of the pedal withdrawal reflex, a laparotomy was performed to collect the urinary bladder. After collecting the urinary bladder, euthanasia was immediately performed by cardiac puncture to ensure complete loss of consciousness and to minimize pain and distress. The collected urinary bladder was carefully excised, weighed, and dissected into rectangular strips approximately 0.5 × 1 cm in size. These tissue samples were immediately transferred to organ baths containing 20 mL of Krebs solution (in mM: NaCl 119, KCl 4.5, CaCl_2_ 2.5, MgSO_4_ 2.5, KH_2_PO_4_ 1.2, NaHCO_3_ 25, glucose 11.1), continuously oxygenated and maintained at 37 °C. An initial resting tension of approximately 2 g was applied to the bladder strips, followed by a 20-min equilibration period.

Contractile responses were initially stimulated by a depolarizing concentration of KCl (80 mM) for 5 min to test tissue viability. The tissues were then washed with Krebs solution, and spontaneous contractile activity of the bladder tissues was recorded for 15 min as a baseline activity (N number: Control = 8; WAS = 8; WAS+2.5KT = 8; WAS+5KT = 8; Soli = 6). Subsequently, cumulative concentrations of carbachol (CCh) (Sigma-Aldrich, Darmstadt, Germany) at 1, 3, 10, and 30 μM were applied to assess bladder contractility in response to muscarinic receptor activation (N number: Control = 7; WAS = 6; WAS+2.5KT = 8; WAS+5KT = 8; Soli = 5). To investigate the contribution of 5-HT_3_ receptors in bladder contractility, the tissues were washed and pre-incubated with ondansetron (30 nM) for 20 min before repeating the carbachol dose–response protocol (N number: Control = 7; WAS = 6; WAS+2.5KT = 8; WAS+5KT = 8; Soli = 5). Bladder contractile parameters, including tone, amplitude, and frequency, were continuously monitored and digitally recorded using the PowerLab® data acquisition system (AD Instruments, Australia), and analyzed with LabChart software version 7.0 (AD Instruments, Chalgrove, UK).

### 2.8 Toluidine blue staining for mast cells

Urinary bladder tissues from all experimental groups were fixed in 10% formalin and subsequently dehydrated through graded ethanol solutions (70%, 95%, and 100%). The samples were subsequently embedded in paraffin and section into 5 μm-thick slices. Mast cells were visualized using 1% toluidine blue staining (Merck, Darmstadt, Germany). Non-degranulated mast cells were identified by the absence of granule extrusion, whereas degranulated mast cells were characterized by the presence of extruded granules. For each animal, three tissue sections were randomly selected for analysis. The number of mast cells in each section was counted, and the mean value was calculated for each mouse. In addition, the percentage of degranulated mast cells in the urinary bladder was determined. Mast cell identification and quantification were performed in a blinded manner using a light microscope (Optika, B-383PLi, Ponteranica BG, Italy) at magnifications of 10 × and 40 × (N number: Control = 6; WAS = 6; WAS+2.5KT = 6; WAS+5KT = 6; Soli = 6).

### 2.9 Statistical analysis

All data are presented as the mean ± standard error of the mean (SEM). Statistical analyses were performed using one-way or two-way ANOVA, followed by Dunnett’s *post hoc* test for multiple comparisons. A *p*-value of < 0.05 was considered statistically significant. All analyses were conducted using GraphPad Prism version 9.0 (GraphPad Software, San Diego, CA, USA).

## 3 Results

### 3.1 Effects of kratom leaf extract administration on body weight and bladder weight in WAS-induced mice

Body weight was measured daily throughout the experimental period. No significant differences in body weight were observed among the experimental groups compared with the control group from day 0 to day 10 (Table 1). After euthanasia, the urinary bladders were collected and weighed. There were no statistically significant differences in bladder weight, expressed as a percentage of body weight, among the experimental groups compared with the control group (Table 1).

**Table 1:** Body weight and the percentage of bladder weight relative to body weight in each group of mice exposed to water avoidance stress for 10 days.

Groups	Body weight (g)		% Bladder weight/ body weight
	Day 0	Day 10	
Control	24.89 ± 1.09	25.68 ± 0.95	0.122 ± 0.007
WAS	24.42 ± 0.43	25.29 ± 0.43	0.130 ± 0.007
WAS + 2.5KT	26.22 ± 0.67	26.40 ± 0.75	0.121 ± 0.004
WAS + 5KT	25.18 ± 1.04	25.51 ± 1.08	0.139 ± 0.012
WAS + Soli	25.20 ± 0.81	26.14 ± 0.70	0.138 ± 0.014

All parameters were analyzed using one-way ANOVA with Dunnett’s multiple comparisons vs. Control group, ns, not significant, *N* = 5–8 in each group.

### 3.2 Effects of kratom leaf extract administration on locomotor activity and anxiety-related behaviors in WAS-induced mice

**Figure 3:**
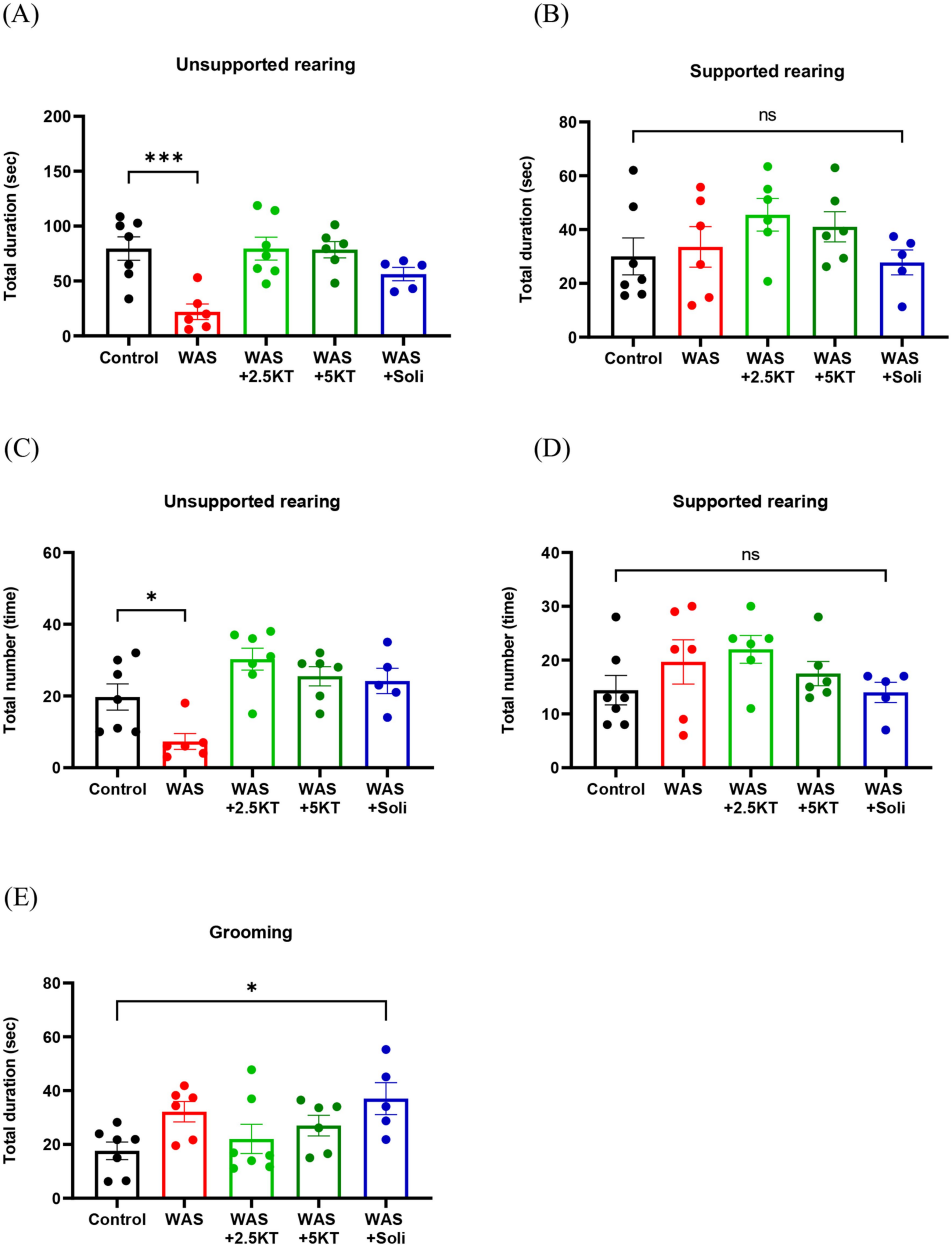
Effects of kratom leaf extract on anxiety-related behavior in mice exposed to water avoidance stress for 10 days. Bar graphs show the total duration **(A,B)** and number **(C,D)** of unsupported and supported rearing, and the total duration of grooming behavior **(E)**. (*p < 0.05, ***p < 0.001, one-way ANOVA with Dunnett’s multiple comparisons vs. control group, ns = not significant, *N* number: control = 7; WAS = 6; WAS+2.5KT = 7; WAS+5KT = 6; Soli = 5).

The effects of kratom leaf extract on anxiety-related behaviors, including unsupported rearing, supported rearing, and grooming, were evaluated. Mice exposed to WAS for 10 days without kratom extract treatment exhibited a significant decrease in both the total duration and total number of unsupported rearings compared with the control group (*p* < 0.001 and *p* < 0.05, respectively) (Figures 3A,C). In contrast, no significant differences were observed in the WAS+2.5KT and WAS+5KT groups compared with the control group (Figures 3A,C). There were no significant differences in either the total duration or the number of supported rearings among the experimental groups compared with the control group (Figures 3B,D). No significant changes in the total duration of grooming were observed among the experimental groups compared with the control group. However, the solifenacin-treated group showed a significant increase in grooming duration (*p* < 0.05) (Figure 3E). As shown in Table 2, there were no significant differences in locomotor activity, including average speed (cm/s), total distance traveled (cm), and time spent in the center zone (s), among the experimental groups compared with the control group.

**Table 2:** Effect of kratom leaf extract on locomotor activity in mice exposed to water avoidance stress.

Groups	Locomotor parameters		
	Averaged speed (cm/s)	Total distance traveled (cm)	Time spent in the center zone (sec)
Control	4.68 ± 0.33	2,768 ± 188.20	152.30 ± 40.90
WAS	4.60 ± 0.20	2,760 ± 119.10	104.20 ± 11.62
WAS + 2.5KT	5.32 ± 0.20	3,179 ± 115.40	91.95 ± 6.41
WAS + 5KT	3.81 ± 0.29	2,279 ± 169.70	131.50 ± 29.16
WAS + Soli	4.83 ± 0.31	2,893 ± 190.70	214.90 ± 30.90

All parameters were analyzed using one-way ANOVA with Dunnett’s multiple comparisons vs. Control group, ns, not significant, *N* = 5–8 in each group.

### 3.3 Effects of kratom leaf extract administration on bladder pain in WAS-induced mice

**Figure 4:**
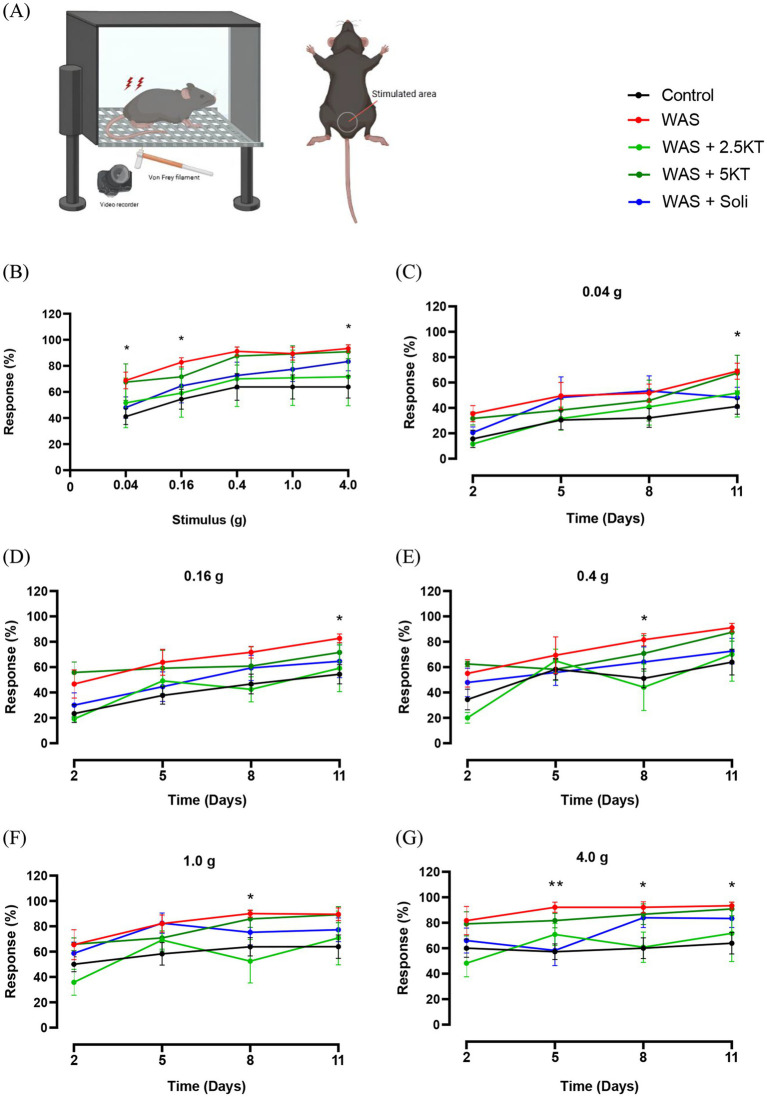
Effects of kratom leaf extract on bladder pain behavior. The illustration depicts an experiment assessing bladder pain sensitivity, where mechanical stimulation was applied to the suprapubic region using von Frey filaments while the mouse was placed in a chamber with a mesh floor **(A)**. Line graphs represent percentages of pain response tested by von Frey filaments (0.04, 0.16, 0.4, 1.0, and 4.0 g) in mice exposed to water avoidance stress for 10 days at day 11 **(B)** and percentages of pain response tested by 0.04, 0.16, 0.4, 1.0, and 4.0 g von Frey filaments on day 2, 5, 8, and 11 **(C–G)**. (*p < 0.05, **p < 0.01, two-way ANOVA with Dunnett’s multiple comparisons vs. ontrol group, ns, not significant, *N* number: control = 6; WAS = 6; WAS+2.5KT = 4; WAS+5KT = 4; Soli = 5).

Bladder pain responses were assessed using von Frey filaments, as illustrated in Figure 4A. On the day following completion of the WAS protocol, the WAS group exhibited a significant increase in pain responses when tested with the 0.04 g, 0.16 g, and 4.0 g filaments compared with the control group (*p* < 0.05) (Figure 4B). In contrast, WAS-induced mice treated with kratom leaf extract (2.5KT and 5KT) or solifenacin showed no significant differences in pain responses at any filament force compared with the control group (Figure 4B). Moreover, analysis of the time course of bladder pain responses revealed that the onset of increased responses in the WAS group varied depending on filament force (Figures 4C–G). Significant increases in pain responses were observed for the 0.04 g and 0.16 g filaments on day 11 (*p* < 0.05), for the 0.4 g and 1.0 g filaments on day 8 (*p* < 0.05), and for the 4.0 g filaments on day 5 (*p* < 0.01). By contrast, pain responses in mice treated with either dose of kratom leaf extract or solifenacin did not differ from those in the control group. These findings indicate that the temporal progression of hypersensitivity varied according to the increasing filament force.

### 3.4 Effects of kratom leaf extract administration on urine voiding patterns in WAS-induced mice

**Figure 5:**
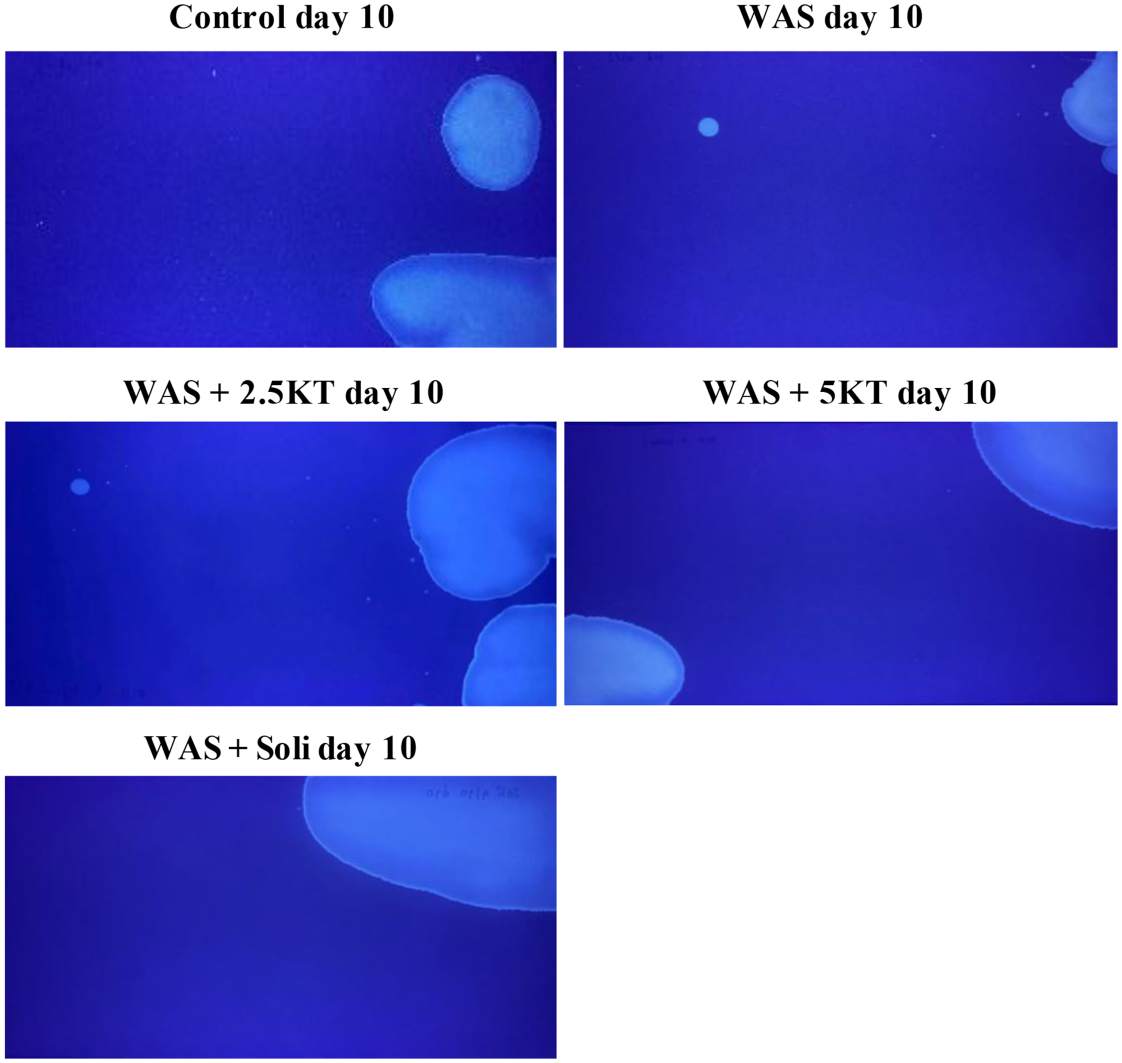
Each experimental group on day 10. The images were captured under UV light to visualize urine spots stained.

**Figure 6:**
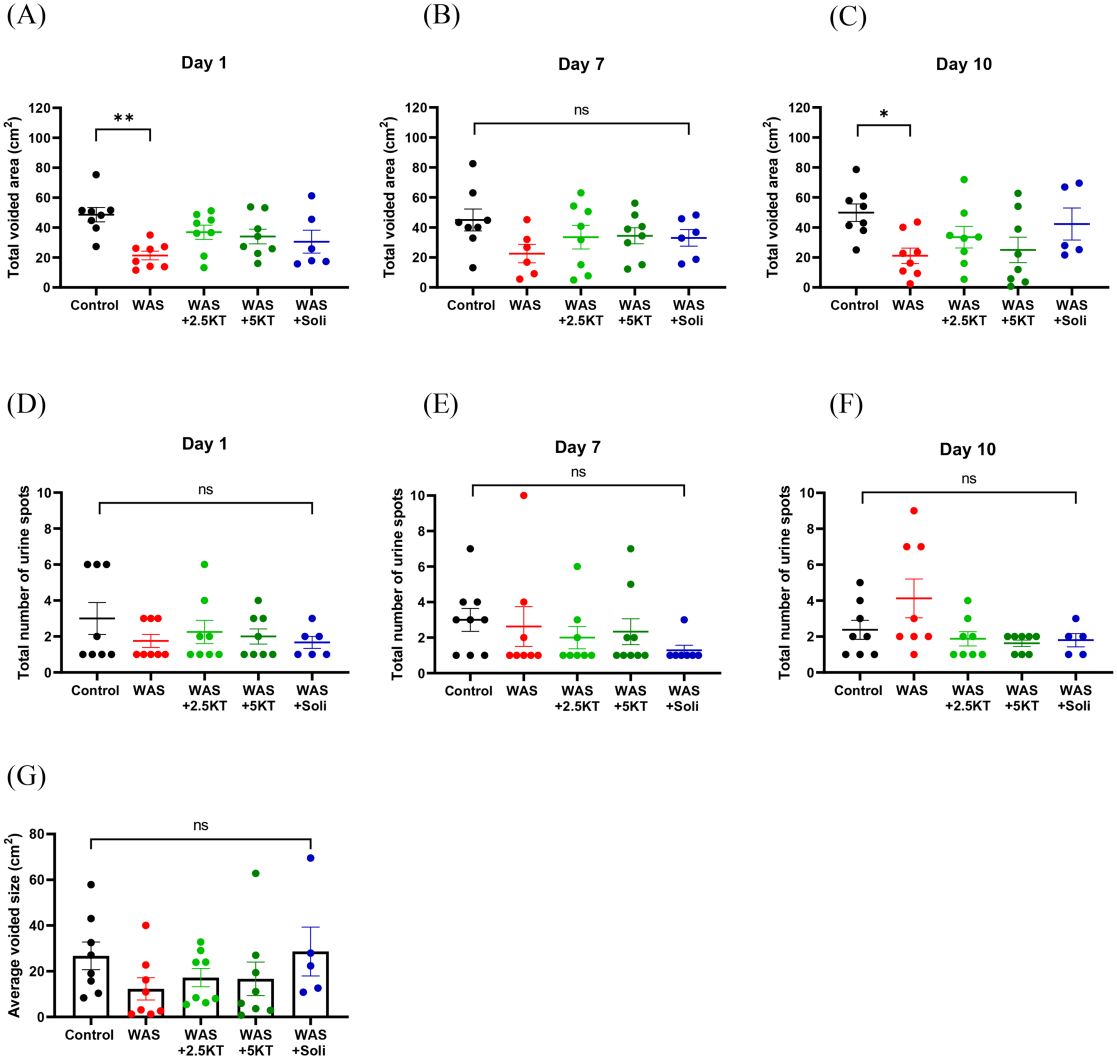
Effects of kratom leaf extract on urine voiding patterns on days 1, 7, and 10 of the WAS protocol. Dot plots with mean ± SEM represent the total voided area **(A–C)**, the total number of urine spots **(D–F)**, and the average voided size on day 10 **(G)**. (*p < 0.05, **p < 0.01, one-way ANOVA with Dunnett’s multiple comparisons vs. control group, ns = not significant, *N* number: control = 8; WAS = 8; WAS+2.5KT = 8; WAS+5KT = 8; Soli = 6).

Figure 5 shows representative images of urine voiding patterns under UV light, illustrating changes in voiding patterns on day 10 for each group. The total voided area and the number of urine spots from voiding spot analysis on days 1, 7, and 10 are presented for all groups, as shown in Figures 6A–F. On Day 1, the WAS exposure group exhibited a significant reduction in the total voided area compared to the control group (*p* < 0.01) (Figure 6A), and treatments with kratom extract (2.5KT and 5KT) or solifenacin showed no statistical changes in voiding area compared with the control group (Figure 6A). On Day 7, no significant differences were observed in the total voided area across all groups (Figure 6B). By day 10, the WAS group continued to show a significantly reduced total voided area compared to controls (*p* < 0.05) (Figure 6C). Treatment with kratom extract (2.5KT and 5KT) and solifenacin tended to restore the voided area in chronic WAS, as no statistically significant differences were found between the treatment groups and the control group (Figure 6C). Regarding the total number of urine spots, no significant differences were observed across the groups on days 1, 7, and 10 compared to the control group (Figures 6D–F). Moreover, there were no significant differences observed in average voided size among the groups on day 10. However, the WAS group displayed a tendency toward reduced average spot sizes compared to the control group (Figure 6G).

### 3.5 Effects of kratom leaf extract administration on bladder contractile properties in WAS-induced mice

**Figure 7:**
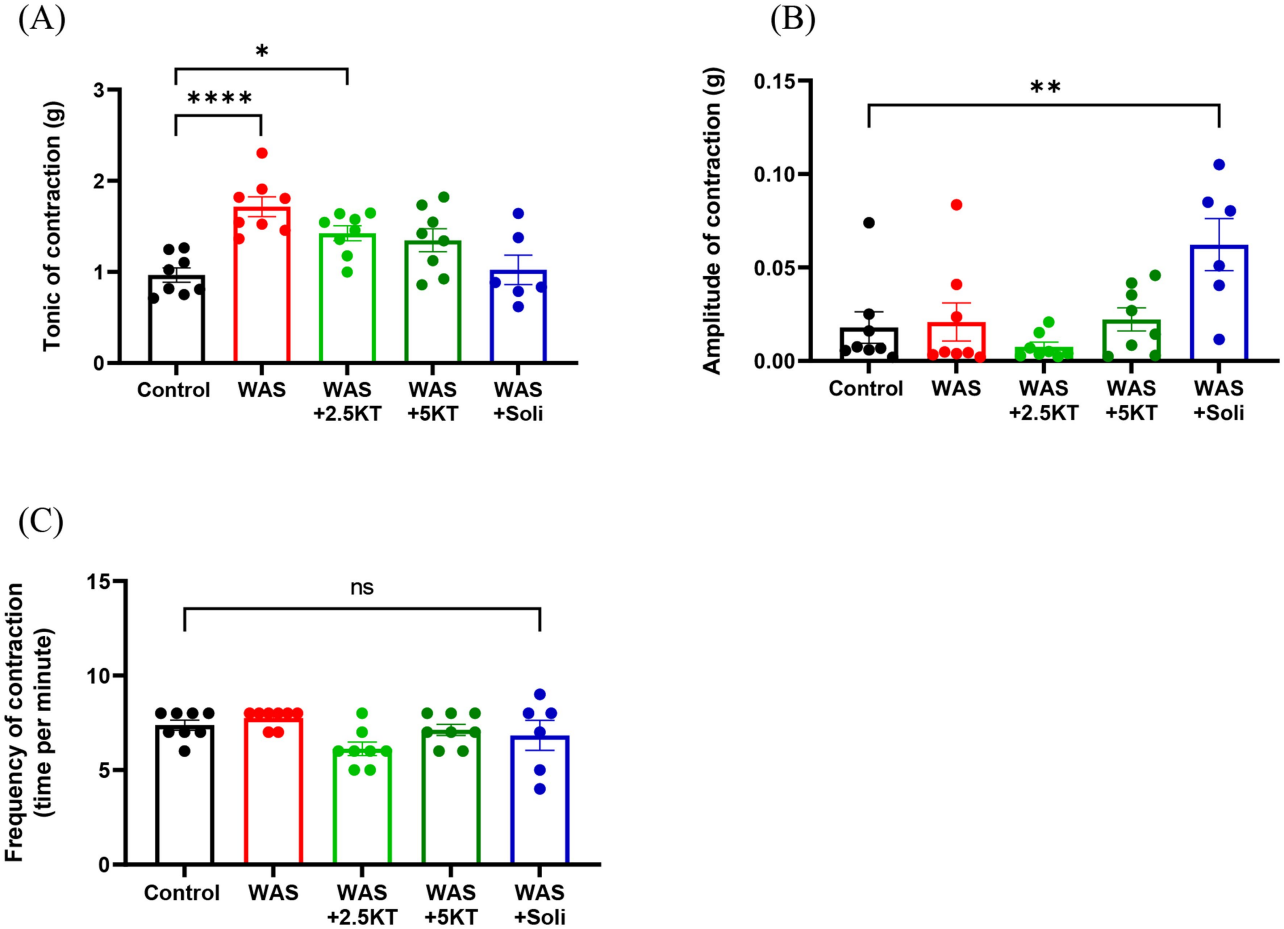
Effects of kratom leaf extract on spontaneous contractile activity of urinary bladder strips. Tonic of contraction **(A)**, amplitude of contraction **(B)**, and frequency of contraction **(C)** of urinary bladder strips after 80 mM KCl-induced contraction. (****p < 0.0001, one-way ANOVA with Dunnett’s multiple comparisons vs. control group, ns, not significant, *N* number: control = 8; WAS = 8; WAS+2.5KT = 8; WAS+5KT = 8; Soli = 6).

To assess spontaneous contractile activity, the tone, amplitude, and frequency of contractions in bladder tissues were examined following 80 mM KCl stimulation. The WAS group exhibited a significant increase in tonic contraction compared to the control group (*p* < 0.0001) (Figure 7A). Notably, the WAS+5KT and WAS+solifenacin groups did not differ significantly from the control group, whereas the WAS+2.5KT group still exhibited a significantly higher tonic contraction (*p* < 0.05) (Figure 7A). The amplitude of contraction was significantly increased in the solifenacin-treated group compared to the control group (*p* < 0.05) (Figure 7B), while no significant changes were detected in the other treatment groups. Regarding contraction frequency, no significant differences were observed among the experimental groups compared to the control group (Figure 7C).

**Figure 8:**
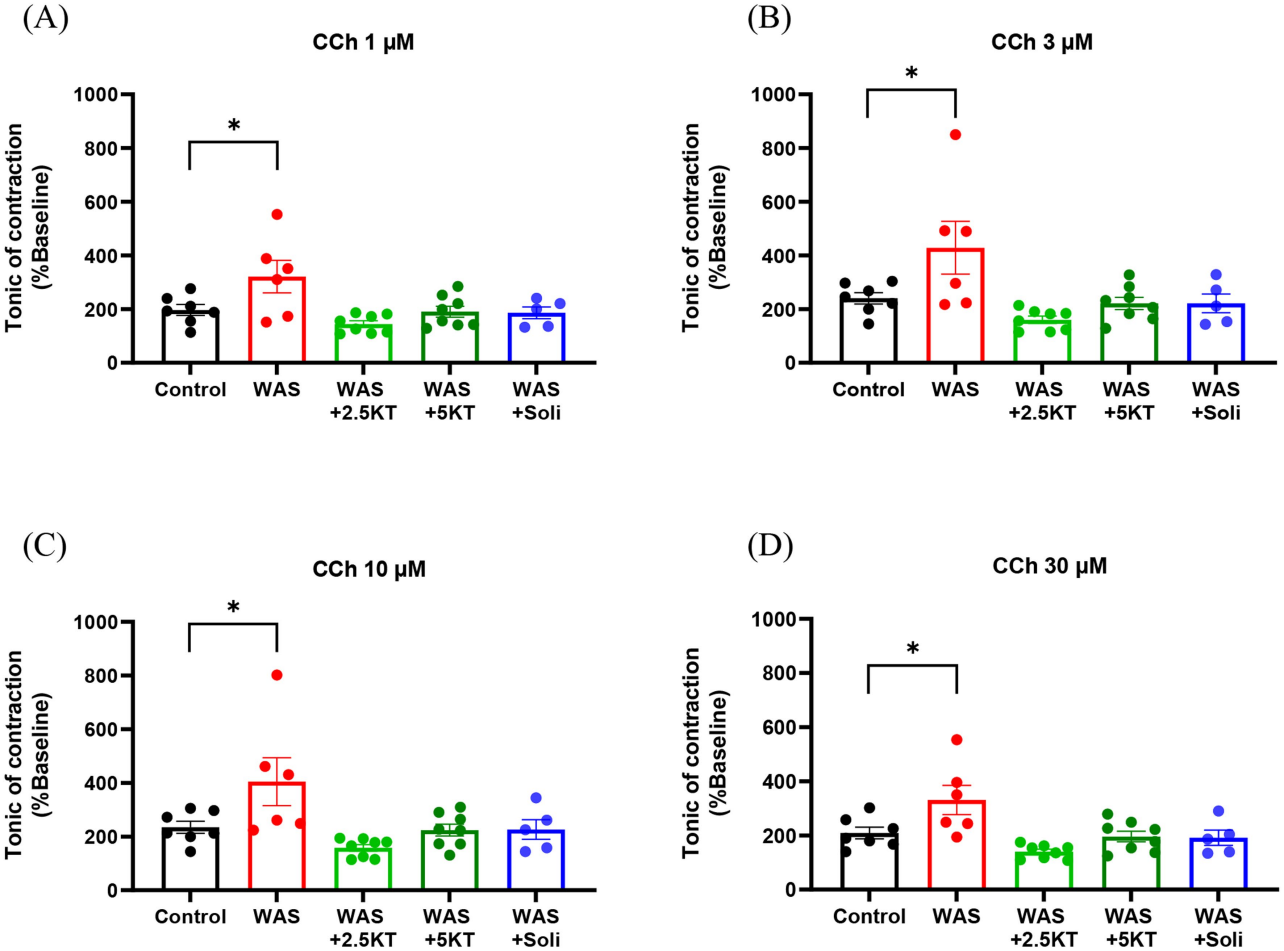
Effects of kratom leaf extract on tonic contraction of urinary bladder strips in response to 1, 3, 10, and 30 μM carbachol (CCh) in mice exposed to water avoidance stress for 10 days **(A–D)**. (*p < 0.05, one-way ANOVA with Dunnett’s multiple comparisons vs. Control group, ns = not significant, *N* number: Control = 7; WAS = 6; WAS+2.5KT = 8; WAS+5KT = 8; Soli = 5).

In addition, the tonic contractile response of the urinary bladder to cumulative concentrations of carbachol (CCh, 1, 3, 10, and 30 μM) was evaluated. The WAS group exhibited a significant increase in tonic contraction of bladder strips across all concentrations compared to the control group (*p* < 0.05) (Figures 8A–D). In contrast, mice treated with kratom leaf extract (2.5KT and 5KT) or solifenacin showed no significant changes in tonic contraction at any CCh concentration (Figures 8A–D). These results suggest that kratom leaf extract mitigates water avoidance stress-induced bladder hypercontractility.

**Figure 9:**
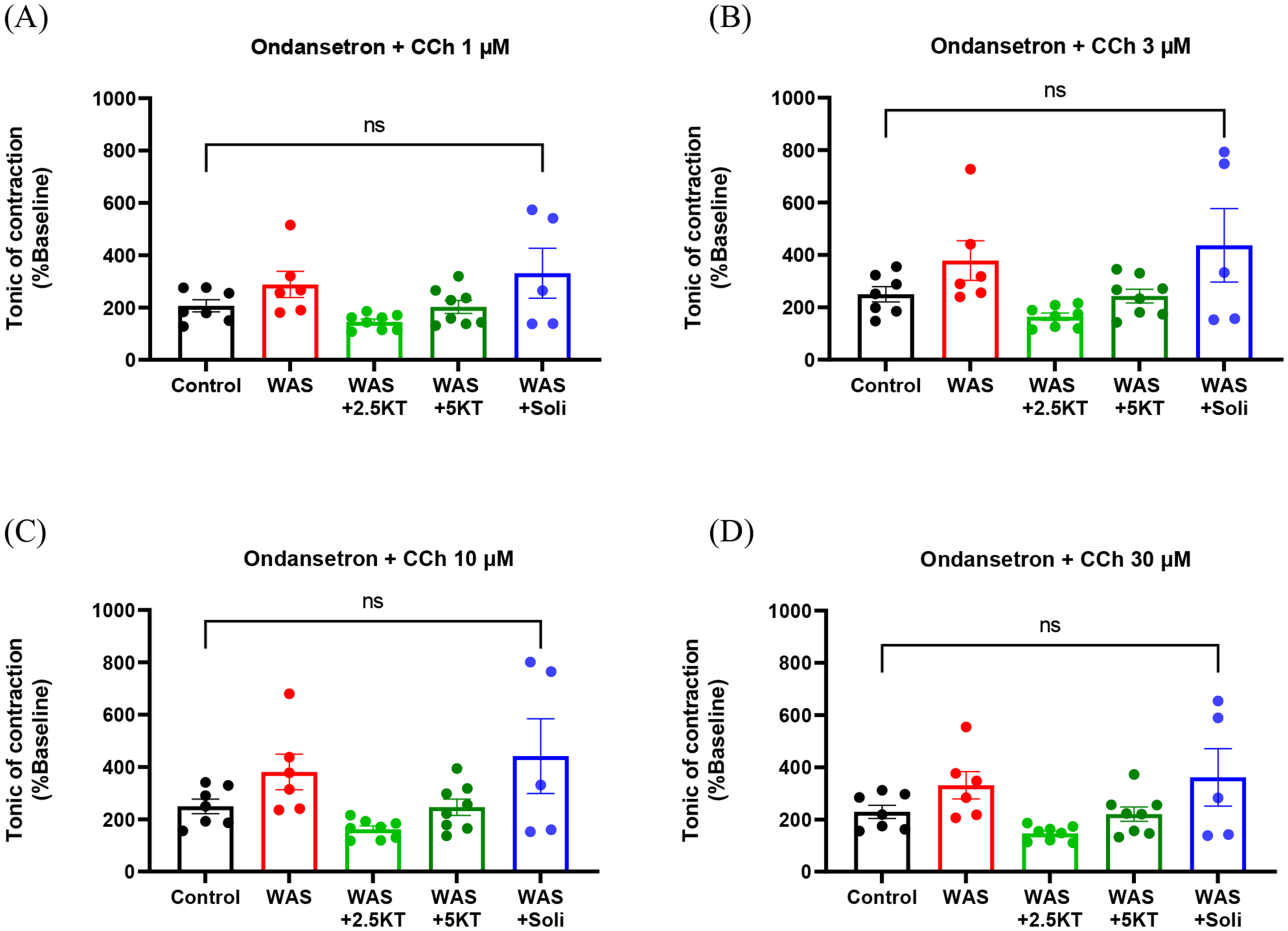
Effects of kratom leaf extract on tonic contraction of urinary bladder strips in response to 1, 3, 10, and 30 μM carbachol (CCh) with ondansetron (30 nM) pre-incubation in mice exposed to water avoidance stress for 10 days **(A–D)**. (*p < 0.05, one-way ANOVA with Dunnett’s multiple comparisons vs. control group, ns, not significant, *N* number: Control = 7; WAS = 6; WAS+2.5KT = 8; WAS+5KT = 8; Soli = 5).

Furthermore, pre-incubation with ondansetron, a 5-HT_3_ receptor antagonist (30 nM), prior to CCh stimulation was also investigated. No statistically significant differences in tonic contraction were observed at any CCh concentration between the treatment and control groups (Figures 9A–D).

### 3.6 Effects of kratom leaf extract administration on mast cell numbers in urinary bladder in WAS-induced mice

**Figure 10:**
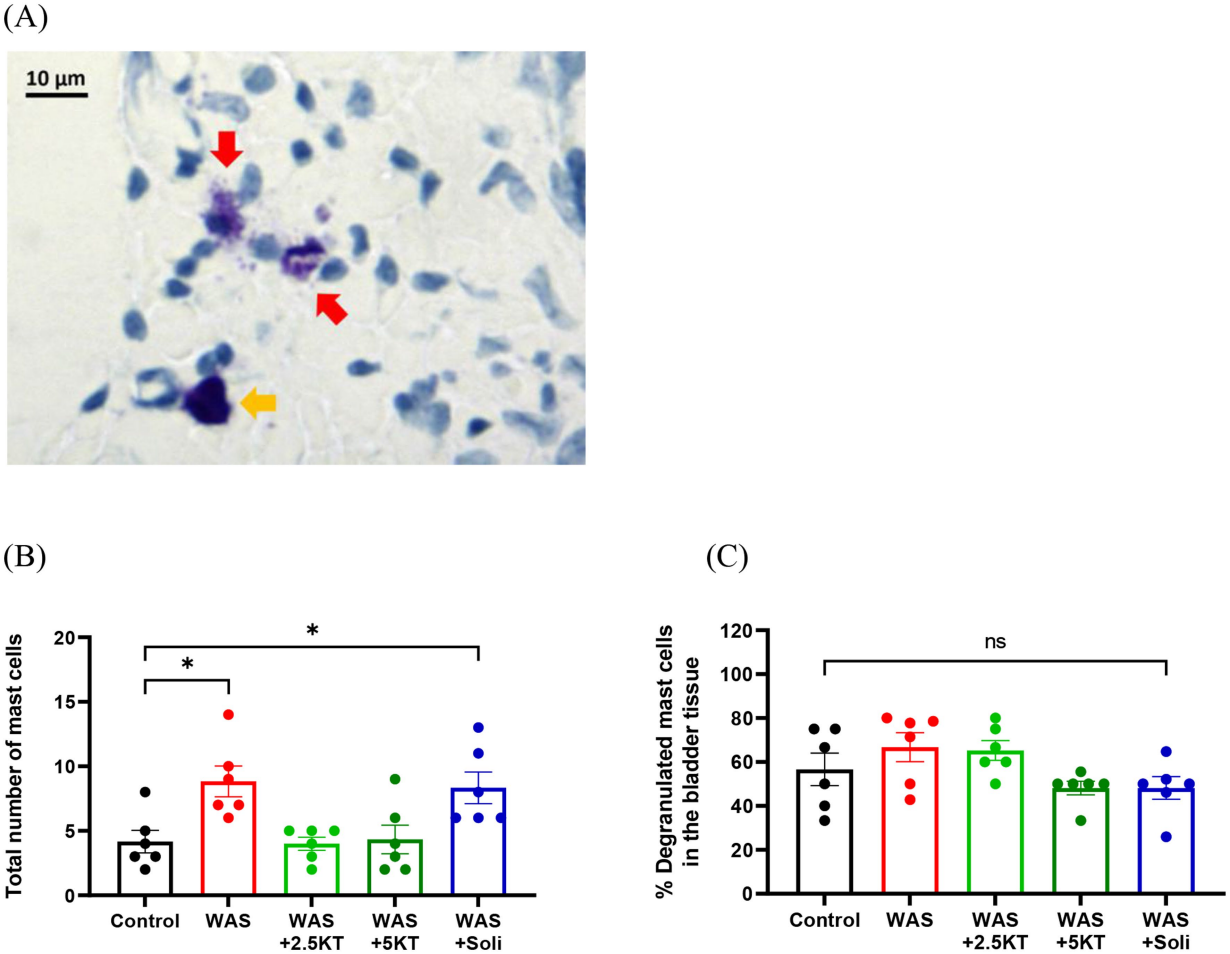
Effects of kratom leaf extract on mast cell infiltration in urinary bladder tissue in mice exposed to water avoidance stress for 10 days. Representative images of degranulated mast cells (red arrow) and non-degranulated mast cell (yellow arrow) **(A)**. Bar graphs represent total number of mast cells **(B)** and percentages of degranulated mast cells in urinary bladder in mice exposed to water avoidance stress for 10 days **(C)**. (*p < 0.05, one-way ANOVA with Dunnett’s multiple comparisons vs. control group, ns, not significant, *N* number: control = 6; WAS = 6; WAS+2.5KT = 6; WAS+5KT = 6; Soli = 6).

Figure 10A shows representative histological images of degranulated and non-degranulated mast cells in the urinary bladder. The WAS and WAS+Solifenacin groups exhibited a significant increase in the total number of mast cells compared to the control group (*p* < 0.05) (Figure 10B). In contrast, mice treated with kratom leaf extract (2.5KT and 5KT) showed no significant changes (Figure 10B). No statistically significant differences were observed among the treatment groups in the percentage of degranulated mast cells in the urinary bladder (Figure 10C).

## 4 Discussion

This study is the first investigation to demonstrate the potential of kratom leaf extract treatment in water avoidance stress-induced anxiety-like behavior, urinary bladder pain, and urinary bladder dysregulation. Our results showed that body weight and normalized bladder weight remained unchanged among the treatment groups. These findings are consistent with previous studies reporting that rats treated with kratom decoction (150 mg/kg) for 28 days showed no significant changes in body weight and hematological parameters (Hassan et al., 2023). Similarly, a 28-day administration of kratom extract (300 mg/kg) in rats did not affect body weight and hematological profiles (Thongsepee et al., 2025). However, our study administered doses of kratom leaf extract, which were determined from the amount of mitragynine, 2.5 and 5 mg/kg in the kratom leaf extract, did not produce addiction side effects in the animals. A study showed that mitragynine induced a preference for the drug-paired compartment in rats at doses starting from 30 mg/kg, while lower doses below 10 mg/kg did not produce conditioned place preference, suggesting a safer profile with lower abuse potential (Meepong and Sooksawate, 2009). A recent study in mice suggests that kratom alkaloids alter dopamine release in the nucleus accumbens, but their effects may not be consistent with those of classic drugs of abuse (Manus et al., 2025).

Our results demonstrated marked alterations in anxiety-related behaviors following chronic WAS exposure, including decreases in both the total duration and total number of unsupported rearing behaviors. However, no significant changes in locomotor activity were observed across the experimental groups, indicating that neither water avoidance stress nor kratom leaf extract administration (2.5 and 5 mg/kg of mitragynine) affected general motor function. This finding is consistent with previous studies (Sattayachiti et al., 2024; Waemong et al., 2024) and suggests that the observed behavioral alterations were independent of changes in locomotor activity. Mice subjected to 10-day WAS exhibited significant reductions in both the duration and frequency of unsupported rearing, an exploratory behavior known to be sensitive to psychological stress. In addition, previous studies have shown stress-induced suppression of exploratory behaviors (Sturman et al., 2018; Concetti et al., 2024). A recent clinical study showed that anxiety can precipitate the onset of OAB by activating oxidative stress and the NF-κB signaling pathway (Zhou et al., 2026). Notably, WAS-exposed mice treated with kratom leaf extract of 2.5 and 5 mg/kg mitragynine showed no significant differences in anxiety-related behaviors compared with control mice. This anxiolytic-like effect is consistent with previous studies demonstrating that intraperitoneal administration of mitragynine significantly reduced immobility time in both the forced swimming test and tail suspension test, with efficacy comparable to conventional antidepressants such as fluoxetine and amitriptyline (Idayu et al., 2011). Moreover, mitragynine treatment has been reported to attenuate plasma corticosterone levels in stress animals, implicating modulation of the hypothalamic–pituitary–adrenal (HPA) axis (Idayu et al., 2011). Besides its interaction with opioid receptors, mitragynine may exert anxiolytic- and antidepressant-like effects through the modulation of multiple neurotransmitter systems. Previous studies have reported anxiolytic-like effects in rats following acute oral administration of mitragynine, which were mediated by GABAergic, dopaminergic, and opioidergic pathways (Hazim et al., 2014). In contrast, solifenacin-treated mice, used as a bladder-targeting positive control, showed an increased grooming duration, which is considered an indicator of anxiety-related behavior. This response is likely attributable to indirect or peripheral effects associated with bladder modulation, consistent with the bladder-selective pharmacological profile of solifenacin (Simpson and Wagstaff, 2005; Chapple et al., 2006), rather than direct alterations in anxiety-related behaviors.

In the present study, we assessed pelvic nociceptive-like behavior using von Frey filament test. Our results demonstrated that WAS significantly enhanced bladder pain responses in mice compared to controls. These findings correlate with previous studies reporting that rats subjected to WAS protocol exhibited a decreased mechanical pain threshold and an increased response frequency to suprapubic stimulation, which are indicative of bladder hyperalgesia (Lee et al., 2015; Matos et al., 2017). Notably, mice treated with kratom leaf extract at doses of 2.5 and 5 mg/kg mitragynine reversed elevated bladder pain responses to von Frey filament following WAS exposure, indicating that the extract attenuated psychological stress–induced pelvic nociceptive sensitization. Many studies indicate that mitragynine exhibits antinociceptive effects, demonstrating its ability to reduce pain perception at various levels of the nervous system (Shaik Mossadeq et al., 2009; Annuar et al., 2024). Both *in vivo* and *in vitro* studies have revealed that mitragynine exerts its antinociceptive actions primarily through activation of the *μ*-opioid receptor, rather than the *δ*- and *κ*-opioid receptors (Suhaimi et al., 2016; Váradi et al., 2016). One possible mechanism of kratom to relieve bladder pain is that mitragynine and its active metabolite, 7-hydroxymitragynine, bind to μ-opioid receptors, which further inhibit the release of neurotransmitters involved in pain signal transmission, such as substance P and calcitonin gene-related peptide (CGRP), thereby directly leading to a reduction in pain perception and producing analgesia (Matsumoto et al., 1996; Matsumoto et al., 2004; Matsumoto et al., 2006; Matsumoto et al., 2006; Beaudry et al., 2011; Kruegel et al., 2019). Importantly, the effects observed following oral administration of mitragynine and 7-hydroxymitragynine were reversed by naloxone, an opioid receptor antagonist, further supporting the role of opioid receptor-dependent mechanisms (Matsumoto et al., 1996; Matsumoto et al., 2004). Moreover, a recent study reported that progressive depletion of functional μ-opioid receptors using methocinnamox preferentially attenuated several 7-hydroxymitragynine-induced antinociceptive effects, supporting the conclusion that opioid receptor signaling is a major contributor to these antinociceptive actions (Brown et al., 2026). An observation of kratom uses in humans suggest short-term pain relief; however, its long-term efficacy and safety remain unclear and require further study (Mun et al., 2025; Waloejo et al., 2026).

In addition to opioid-mediated actions, mitragynine’s antinociceptive properties may also involve non-opioid pathways, particularly adrenergic signaling (Swogger et al., 2022). Pharmacological evidence suggests that mitragynine acts as an antagonist at various *α*-adrenoceptor subtypes (Obeng et al., 2020). In animal models, systemic administration of α-adrenergic agonists has been shown to provoke bladder pain, which can be alleviated through selective blockade of α₁A-adrenoceptors (Matos et al., 2016). Furthermore, activation of peripheral α_1_A-adrenoceptors has been implicated in the development of chronic visceral pain by sensitizing TRPV1 receptors and increasing ATP release (Matos et al., 2016). Moreover, previous investigations demonstrated that WAS exposure in rodents elevates urinary noradrenaline levels and bladder pain sensitivity, both of which are suppressed by the α_1_A-adrenoceptor antagonist, silodosin (Matos et al., 2017). Animal studies suggest that kratom may have beneficial effects on pain, anxiety, and depression, likely through modulation of opioid, noradrenergic, serotonergic, and dopaminergic systems (Green et al., 2025). However, further studies are necessary to prove the related mechanism of kratom leaf extract and mitragynine in exerting analgesic effect in the urinary bladder in this model.

The WAS animal model is also well established to induce impairments in voiding function, including increased urinary frequency, a higher number of small urine spots, and reductions in average voided volume and total urine output (West et al., 2021a; Sattayachiti et al., 2024), reflecting features consistent with the overactive bladder phenotype. In the present study, although the total number of urine spots did not differ significantly, mice exposed to WAS exhibited a trend toward increased urinary frequency and reduced total voided area, indicating decreased urine volume per void. Our findings are consistent with several recent studies showing that chronic stress can induce OAB-like symptoms, including increased urinary frequency and reduced average void volume (Husain et al., 2025b). Prolonged activation of neuroendocrine stress pathways, accompanied by BDNF overexpression in the paraventricular nucleus of the hypothalamus (PVN), markedly decreased intermicturition intervals and voided volumes, reduced bladder capacity, and caused bladder wall hypertrophy (Husain et al., 2025a). To further assess functional consequences, bladder contractile properties were examined using organ bath experiments. Bladders from WAS-exposed mice displayed spontaneous hypercontractility, particularly in tonic contractions. Tonic contractions of urinary bladder strips in response to increasing concentrations of carbachol, a non-selective cholinergic agonist mimicking acetylcholine-mediated contraction, were significantly enhanced in the WAS group compared to controls. These findings align with a previous report showing elevated spontaneous bladder contractions in both tone and amplitude following stimulation with a high concentration of KCl and enhanced response to carbachol in WAS-induced mice (Sattayachiti et al., 2022). These results are consistent with prior studies indicating that WAS exposure increases bladder contractile responses to muscarinic, purinergic, and KCl stimulation (West et al., 2021a). Moreover, muscarinic signaling has been shown to raise detrusor overactivity under stress conditions via upregulation of M_3_ muscarinic receptors on the bladder (Imamura et al., 2014), highlighting the pivotal role of muscarinic pathways in stress-induced bladder dysfunction. Collectively, these findings suggest that psychological stress disrupts voiding patterns and enhances bladder contractility, likely through muscarinic signaling-mediated hyperexcitability. Additionally, our results also showed that pre-incubation with ondansetron, a selective 5-HT_3_ receptor antagonist, abolished the differences in carbachol-induced contractions observed in the WAS group. This finding indicates that serotonergic signaling via 5-HT_3_ receptors acts as a critical modulator of stress-enhanced cholinergic bladder responses (Sattayachiti et al., 2022). However, one of the limitations of this investigation is that it was conducted only in male mice; the observed responses may differ in female mice.

Administration of solifenacin, a selective muscarinic receptor antagonist, effectively improved total voided area in WAS-exposed mice, consistent with its clinical efficacy in patients with overactive bladder (West et al., 2022b; Chapple et al., 2004). These results support that muscarinic receptor blockade can ameliorate stress-induced bladder dysfunction. However, its clinical use is frequently associated with adverse effects such as dry mouth, constipation, and blurred vision, which may limit long-term use (Chapple et al., 2006). Interestingly, our results demonstrate that kratom leaf extract improved voiding patterns and modulated bladder contractility in WAS-exposed mice. Kratom treatment significantly corrected the total voided area and the number of urine spots in the voiding spot assay. In addition, treatment with kratom leaf extract at 5 mg/kg mitragynine effectively alleviated the WAS-induced increase in tonic spontaneous contractions. Notably, kratom leaf extract reversed bladder hypercontractility in response to a muscarinic agonist, carbachol. A recent study on acetic acid-induced colitis showed that kratom leaf extract containing mitragynine suppressed intestinal smooth muscle hypercontractility, including contractions triggered by KCl, carbachol, and serotonin, while promoting smooth muscle relaxation via the mu-opioid agonistic properties of the extract (Pradab et al., 2026). These findings highlight the potential of kratom leaf extract to attenuate stress-induced bladder hypercontractility through reduced contractile response to muscarinic stimulation. However, future studies should be conducted to further elucidate the involvement of muscarinic signaling, including the determination of key enzymes in the pathway or determining gene expression of muscarinic receptors, to ensure the underlying mechanism. In addition to a muscarinic-related mechanism, previous studies have demonstrated that mitragynine inhibits electrically stimulated contractions of isolated guinea-pig ileum in a naloxone-sensitive manner, suggesting involvement of opioid receptor-mediated mechanisms (Watanabe et al., 1997). Mitragynine has also been shown to suppress neurogenic smooth muscle contractions, such as nerve-evoked vas deferens responses, potentially through blockade of neuronal Ca^2+^ channels (Matsumoto et al., 2005). Furthermore, oral administration of kratom extract in rats produces a dose-dependent antidiarrheal effect that is not reversed by naloxone, indicating the contribution of complementary non-opioid mechanisms (Chittrakarn et al., 2008). Collectively, these findings support the therapeutic potential of kratom leaf extract for stress-induced bladder dysfunction and IC/PBS. However, further investigations are necessary to clarify the underlying pathways.

In the present study, mast cell infiltration in the urinary bladder was observed following exposure to the WAS protocol. Kratom treatment appeared to reverse this stress-induced mast cell infiltration, suggesting that the protective effects of kratom leaf extract on bladder dysfunction may involve modulation of inflammatory pathways. Previous studies have demonstrated that kratom exhibits potent anti-inflammatory properties, including suppression of pro-inflammatory cytokines such as TNF-α, IL-6, and inhibition of COX-2 expression (Rahmawati et al., 2024). These inflammatory mediators are known to play a key role in mast cell recruitment, activation, and degranulation, supporting a potential link between kratom’s anti-inflammatory actions and reduced mast cell–associated bladder dysfunction under stress conditions. Psychological stress has been increasingly recognized as a key modulator of bladder function through neuroimmune mechanisms. Activation of the hypothalamic–pituitary–adrenal (HPA) axis during stress leads to the release of mediators such as corticotropin-releasing hormone, which can directly influence immune cell activity, including mast cells. Furthermore, stress-induced release of neuropeptides (e.g., substance P, calcitonin gene-related peptide) from sensory nerves plays a critical role in mast cell activation (Kempuraj et al., 2019), particularly given their close anatomical proximity within bladder tissue. Mast cells serve as key effector cells capable of synthesizing, storing, and releasing an array of inflammatory mediators and neuroactive substances, including serotonin, histamine, prostaglandins, and cytokines (Matos et al., 2017; Herr et al., 2017). Previous studies have shown that stress-induced activation of bladder mast cells can amplify bladder inflammation and sensory hypersensitivity, contributing to the severity of symptoms observed in interstitial cystitis/bladder pain syndrome (IC/BPS) (Smith et al., 2011; Blin et al., 2023). In addition, an experimental study has demonstrated that mast cells contribute to the increased voiding frequency observed following stress exposure (Sidwell et al., 2024). This neuroimmune interaction is proposed to contribute to the development of stress-induced bladder dysfunction; however, further experimental studies are required to elucidate and confirm the underlying mechanisms.

Our study suggests that kratom leaf extract holds potential for the treatment of stress-induced bladder overactivity. However, another limitation of this study is that several other bioactive alkaloids (e.g., 7-hydroxymitragynine, paynantheine, speciogynine) that may contribute to the observed effects, the pure compound of mitragynine should be further investigated. However, our study provides an essential finding about its beneficial effects in using kratom in the form of water extract, which is common and related to the traditional usage of people in the local area. Future studies should therefore elucidate the deep underlying mechanisms of kratom extract or mitragynine to ameliorate stress-induced urinary bladder dysfunction.

## 5 Conclusion

Oral administration of kratom leaf extract containing 2.5 and 5 mg/kg of mitragynine effectively reduced anxiety-like behavior and mitigated bladder pain in the WAS model, while also improving voiding patterns, alleviating bladder hypercontractility in response to muscarinic stimulation, and reducing mast cell infiltration in the urinary bladder under psychological stress. These findings highlight the therapeutic potential of kratom leaf extract for managing stress-related urinary bladder dysfunction.

## Data Availability

The raw data supporting the conclusions of this article will be made available by the authors, without undue reservation.
